# Exposure and Emotional Reactivity to Daily Stressors in Same-Sex and Different-Sex Marriages

**DOI:** 10.1111/jomf.70058

**Published:** 2026-01-26

**Authors:** Michael A. Garcia, Rachel Donnelly, Debra Umberson

**Affiliations:** 1 Department of Sociology and Population Research Center, The University of Texas at Austin, Austin, Texas, USA; 2 Department of Sociology, Vanderbilt University, Nashville, Tennessee, USA

**Keywords:** gender, marital roles, quantitative methodology, same-sex marriage, stress, well-being

## Abstract

**Objective:**

This study examines differences in exposure and emotional reactivity to daily stressors for women and men in same-sex and different-sex marriages.

**Background:**

Prior research on daily stress processes in marriage suggests that women may face heightened exposure and emotional reactivity to daily stressors compared to men. However, prior studies have focused on women and men in different-sex couples, raising questions about whether and how gendered daily stress processes unfold differently for women and men in same-sex marriages.

**Method:**

Ten days of dyadic diary data were used to examine variation in daily stress processes in same-sex and different-sex married couples (*n* = 756 individuals; 378 couples). Mixed effects multilevel modeling was used to estimate individual gender and dyad gender (i.e., same-sex vs. different-sex) effects on (1) exposure and (2) emotional reactivity to daily stressors (i.e., same-day association between daily stressor exposure and daily psychological distress).

**Results:**

Women reported more daily stressors than men, regardless of marriage composition. Emotional reactivity to daily stressors was especially heightened for women in different-sex marriages and, to a lesser extent, women and men in same-sex marriages compared to men in different-sex marriages.

**Conclusion:**

Results highlight the importance of a dyadic perspective and underscore the need to include same-sex couples in studies of gendered daily stress processes in marriage.

Marriage has differential costs and benefits for the well-being of women and men (Kiecolt-Glaser and Newton 2001; Liu and Umberson 2008; Rendall et al. 2011) due, in part, to inequality in daily stress processes (Almeida and Kessler 1998). Daily stressors such as arguments with a spouse, problems at work, and concerns about finances are common occurrences that have immediate implications for spouses’ well-being (Almeida 2005). In addition to the short-term effects of daily stress exposure, greater emotional reactivity (i.e., the likelihood that an individual will respond emotionally to stressors; Almeida 2005; Almeida et al. 2011) can have long-lasting impacts on well-being (Kiecolt-Glaser et al. 2020). In different-sex marriages, women report greater exposure (Villeneuve et al. 2014) and reactivity (Bolger et al. 1989) to daily stressors than men, perhaps due to gender differences in power that produce differential demands related to home and family life (Umberson and Thomeer 2020). Support exchanges also often differ by gender in different-sex marriages, with women more likely to provide support to their spouse and men more likely to withdraw during stressful situations (Falconier and Kuhn 2019; Umberson et al. 2016). Such differences could place women in different-sex marriages at a disadvantage in coping with daily stressors, which may heighten reactivity compared to men.

Gender is clearly key to understanding daily stress processes in different-sex marriages, yet a gender-as-relational approach suggests that gendered expectations and performances often differ depending on whether an individual is interacting with a woman or a man (Connell 2012; Springer et al. 2012). Thus, stress processes may operate differently for men and women in same-sex compared to different-sex relationships. In the present study, we draw on a gender-as-relational perspective and analyze 10 days of diary data from both spouses (i.e., dyadic data) in midlife same-sex and different-sex marriages (*n* = 756 individuals) to examine variation in exposure and emotional reactivity to daily stressors. We expect daily stress processes to differ depending on the individual’s gender, their spouse’s gender, and the gender composition of the couple (i.e., same-sex vs. different-sex).

Studies of gendered daily stress processes, particularly emotional reactivity to stress, in different-sex marriages are scant and in need of updating. Gender differences in emotional reactivity to stressors have not been empirically tested in over 35 years despite significant changes in gender dynamics related to work and home life, which may influence daily stress processes within marriages (Fry et al. 2023; Perry-Jenkins and Gerstel 2020; Ridgeway 2011). This study provides a much-needed update to the empirical literature and further extends this body of work by including long-term, legally married same-sex couples, a growing population that faces inequality in stress exposure (Meyer 2013) and well-being (National Academies of Sciences, Engineering, and Medicine 2020). By expanding our understanding of how daily stress processes unfold in both same-sex and different-sex marriages, this study can inform theories of gendered marital dynamics and identify areas where intervention may promote the well-being of married couples.

## 1 | Background

Marriage has long been considered a gendered institution that produces differential costs and benefits for the health and well-being of women and men (Kiecolt-Glaser and Newton 2001; Liu and Umberson 2008; Rendall et al. 2011). These costs and benefits may be gendered in part because of inequality in daily stress processes (Almeida and Kessler 1998; Bolger et al. 1989). Exposure to daily stressors, or the routine hassles of daily life, can have immediate consequences for well-being (Almeida 2005; Almeida et al. 2002). Indeed, prior research suggests that people report greater psychological distress on days they experience stressors than on stressor-free days (Almeida 2005; Almeida et al. 2011). However, daily stress processes are socially patterned such that women and sexual minorities have a greater risk of exposure and emotional reactivity to daily stressors than men and sexual majorities (Pearlin 2010; Thoits 2010). Accordingly, researchers have called for same-sex couples to be included in studies of marital dynamics to examine gender dynamics within and across union types and to reflect the growing diversity of contemporary marriages (Umberson et al. 2015; West et al. 2008; Thomeer et al. 2020). Focusing solely on different-sex couples obscures the diversity of gendered marital experiences, especially across sexual orientations (Reczek 2020), and limits our understanding of gendered processes in marriage (Thomeer et al. 2020; West et al. 2008).

Thus, the present study uses a gender-as-relational perspective to explore gendered daily stress processes in same-sex and different-sex marriages. This approach frames gender as an ongoing, negotiated process that is institutionalized and stratified to signify power and to structure interactions between and among different genders (Connell 2012; Springer et al. 2012). Prior studies comparing same-sex and different-sex married couples highlight the usefulness of a gender-as-relational approach for understanding daily marital dynamics (see Thomeer et al. 2020). The gender-as-relational approach can challenge taken-for-granted assumptions about gender differences within marriage based on decades of research on different-sex couples. For example, one study found that although daily marital strain undermined daily psychological well-being in both same-sex and different-sex couples, the association was stronger for women in different-sex marriages compared to women in same-sex marriages (Garcia and Umberson 2019). This finding suggests that the combination of individual gender and spouse gender is an important factor in shaping the processes that influence spouses’ daily psychological well-being.

Such an approach is especially important when considering daily stress processes within the context of marriage. Stress can often be “shared” within marriages as stress in one domain can spill over into other domains (Buck and Neff 2012) and cross over from one spouse to the other (Craig and Brown 2017; Cooper et al. 2020). Even when stressors occur outside of the marriage (e.g., conflict at work or with friends), spouses often help each other cope with stressful experiences. Yet, coping efforts tend to differ for men and women such that women are more responsive to their spouse’s needs, more likely to hide their own stress from their spouse, and more engaged in collaborative coping efforts compared to men (e.g., Bodenmann et al. 2006; Erickson 2005; Walen and Lachman 2000). However, these gender dynamics may unfold differently in same-sex marriages. Indeed, same-sex spouses tend to share emotional labor more equitably (Umberson et al. 2015; Wang and Umberson 2023) and receive more support from their spouse (Donnelly et al. 2018) compared to spouses in different-sex marriages. Thus, the present study makes use of the gender-as-relational approach by considering how exposure and emotional reactivity to stressors may differ for women and men in different-sex and same-sex marriages.

### 1.1 | Daily Stressor Exposure in Different-Sex Marriages

In different-sex marriages, women report more frequent exposure to daily stressors than men (Villeneuve et al. 2014), perhaps due to structural power differences that produce different home and family demands for women and men (Umberson and Thomeer 2020). Though the gender gap has narrowed (Van Gorp 2013), women in different-sex marriages continue to bear a heavier burden of unpaid labor than men, including housework (Bianchi et al. 2012), child-care (Tichenor 2005), and emotional labor (Erickson 2005; Umberson et al. 2015). Women in different-sex marriages also shoulder more cognitive labor, an often-invisible form of labor that involves anticipating needs and managing family logistics (Daminger 2019), which has been linked to worse well-being, particularly for women (Petts and Carlson 2023). This unequal burden of household labor is often in addition to paid labor, resulting in a “second shift” wherein women are expected to care for their husbands and children when they come home from their paid work (Hochschild and Machung 2012). Even when divisions of labor are more equitable, women tend to perform tasks that require regular and frequent attention such as cooking and laundry, whereas men tend to handle intermittent tasks such as lawn care and car maintenance (Friedman 2015; Kroska 2003; Roxburgh 2004). This multitude of tasks can lead to an overload of daily stressors for women in different-sex marriages.

### 1.2 | Daily Stressor Exposure in Same-Sex Marriages

Although less is known about how gender shapes daily stressor exposure in same-sex marriages, there are multiple reasons to expect that daily stressor exposure differs for same-sex couples compared to different-sex couples. On the one hand, same-sex couples tend to be more equitable in their divisions of household labor (Goldberg et al. 2012; Tornello et al. 2015), which could result in fewer daily stressors compared with women in different-sex couples who largely shoulder the responsibility for such labor. Conversely, because spouses in same-sex marriages may experience additional individual- and couple-level stressors associated with their sexual minority status (Cao et al. 2017; Frost et al. 2017; Meyer 2013), they could face a greater daily stressor burden than spouses in different-sex marriages. Minority stress theory explicates that sexual minorities experience unique proximal (e.g., concealment) and distal (e.g., discrimination) social stressors that can negatively impact mental health and well-being (Meyer 2013). Moreover, being in a same-sex couple can expose individuals to unique minority stressors in addition to individual-level minority stressors (e.g., public scrutiny, not being perceived as a couple; Frost et al. 2017). As such, men and women in same-sex marriages could experience more daily stressors than spouses in different-sex marriages, especially men in different-sex marriages who are advantaged by both gender and sexual orientation. Taken together, differences in divisions of labor and minority stress processes underscore the need to consider how gender may shape variation in daily stressor exposure across both same-sex marriages and different-sex marriages.

### 1.3 | Emotional Reactivity to Daily Stressors in Different-Sex Marriages

Prior research on different-sex married couples suggests that, in addition to facing greater exposure to daily stressors, women may be more emotionally reactive to daily stressors than men (Bolger et al. 1989), though this hasn’t been tested empirically in recent decades. Daily stressors often “pile up” over a series of days and create patterns of chronic stress that undermine well-being (Almeida 2005; Thoits 2010), and chronic home-related stress exacerbates this negative effect (Serido et al. 2004). If women in different-sex marriages consistently experience an overload of daily stressors, their emotional reactions to such exposure could be heightened relative to men in different-sex marriages.

Differences in support exchanges may also explain gender differences in emotional reactivity to daily stressors within different-sex marriages. Women often suppress their own emotional needs (Thomeer, Umberson, and Pudrovska 2013) and provide more and higher quality support to their spouses than they receive (Umberson et al. 2015), creating a “support gap” between women and men in different-sex marriages (Neff and Karney 2005). This is especially evident during periods of stress. Whereas men report receiving higher quality support from their wives on high-stress days, women report receiving both support and negativity from their husbands (Neff and Karney 2005). Men are also more likely than women to downplay their spouse’s distress (Thomeer, Umberson, and Pudrovska 2013). Thus, for men, support from their wives may reduce the negative consequences of daily stressor exposure, whereas for women, a relative lack of support and an additional burden of negativity from their husbands may exacerbate emotional reactivity to daily stressors.

### 1.4 | Emotional Reactivity to Daily Stressors in Same-Sex Marriages

Scholars have yet to examine emotional reactivity to daily stressors in same-sex marriages. This gap is important to address considering the heightened risk of stress and disadvantaged mental health profiles of sexual minorities (Meyer 2013; National Academies of Sciences, Engineering, and Medicine 2020). Marital communication and support exchanges during times of stress could also lead to differences in emotional reactivity between same-sex and different-sex couples. Compared to different-sex spouses, same-sex spouses communicate with each other more effectively (Rostosky and Riggle 2017) and tend to be more equitable in sharing emotional labor (Umberson et al. 2015; Wang and Umberson 2023), which could leave them better equipped to cope with daily stressors, thereby reducing emotional reactivity to daily stressors. Moreover, same-sex spouses report receiving greater spousal support than different-sex couples during times of stress (Donnelly et al. 2018) and are more likely to cope with stress collaboratively (Wang and Umberson 2023). These factors may lessen reactivity in same-sex couples, especially compared with women in different-sex couples, who are more likely to suffer from a support gap (Neff and Karney 2005).

Alternatively, sharing stress and shouldering the additional burden of minority stressors may exacerbate reactivity, especially compared with men in different-sex marriages who benefit from spousal disparities in emotion work. For example, women in different-sex and same-sex marriages are more strongly influenced by their spouse’s distress compared to men, although this association is particularly strong for women married to men (Behler et al. 2019). Individual-level minority stressors can also spill over into relationships and impact both partners’ well-being (Frost et al. 2017). Indeed, Cooper et al. (2020) show that minority stressors cross over between same-sex spouses in ways that are above and beyond that of general daily stressors, which could heighten reactivity relative to different-sex spouses. Differences in marital communication and support, as well as unique exposures to minority stressors, highlight the need to consider variation in emotional reactivity to daily stressors across and within same- and different-sex marriages.

### 1.5 | Covariates

Prior studies suggest several individual- and couple-level covariates that are associated with stress, marital dynamics, and well-being. That is, individual sociodemographic characteristics (e.g., age, educational attainment) structure exposure to stress and shape patterns of well-being (e.g., Thoits 2010), and relationship characteristics (e.g., relationship duration, marital quality) influence marital dynamics with consequences for health and well-being (e.g., Carr and Utz 2020; Umberson and Thomeer 2020).

### 1.6 | The Present Study

In the present study, we analyze 10 days of dyadic diary data collected from 756 midlife women and men in same-sex and different-sex marriages (*n* = 378 couples) to examine daily stressor exposure (e.g., stressors arising from relationships, work, home, etc.) and emotional reactivity (i.e., same-day association between daily stressor exposure and daily psychological distress) and consider whether these processes vary for women and men in same-sex and different-sex marriages.

Prior research emphasizes the importance of dyadic data in studying marital dynamics as it allows researchers to consider how the characteristics and experiences of each spouse in a marital dyad combine to shape individual- and couple-level functioning (Bolger et al. 1989; Carr and Springer 2010). Because daily stressors fluctuate from day to day, diary studies are particularly well-suited to understanding how people respond to stressors in everyday life (Almeida 2005). Indeed, daily diaries are an innovative method for “capturing life as it is lived” (Bolger et al. 2003) because they enable researchers to assess natural fluctuations in daily stressors and well-being shortly after they occur, unlike traditional survey methods that rely on global evaluations of stress and well-being over longer periods of time (Almeida 2005).

The present sample includes a relatively unique cohort of midlife same-sex couples who chose to marry between 2004 and 2012 when US states began to adopt marriage equality. By the time *Obergefell v. Hodges* established marriage equality in all 50 states in 2015, 36 states had adopted marriage equality. As such, this unique cohort of midlife adults lived through dynamic socio-political changes that likely shape their exposure to stress and their marital dynamics. Moreover, these life course exposures could have lasting impacts on this cohort as they age, pointing to the need for continued study of long-term same-sex couples who established and maintained their relationships in a rapidly evolving landscape. Although it is possible that this cohort may differ from younger cohorts who grew up with more supportive social norms and environments, prior research finds that the improved social environment has not reduced the exposure of younger sexual minority birth cohorts to minority stressors (Meyer et al. 2021). Because prior theoretical and empirical work is mixed, we do not form directional hypotheses about gender differences in exposure and reactivity to daily stressors. Instead, we address the following research questions:

1. To what extent does exposure to daily stressors differ for women and men in same-sex and different-sex marriages?
2. To what extent does emotional reactivity to daily stressors differ for women and men in same-sex and different-sex marriages?

## 2 | Method

### 2.1 | Data and Sample

To examine exposure and emotional reactivity to daily stressors, we use data from the Health and Relationships Project (HARP; Umberson 2019), an ongoing dyadic study of marital dynamics, health, and well-being. HARP is uniquely suited to the present study because it is one of the first studies to include both spouses in long-term, legally married same-sex couples in addition to different-sex couples. Initial data collection occurred from 2014 to 2015. At the time of recruitment, all participants were between the ages of 35 and 65 and had been legally married for at least 3 years. The HARP sample was recruited in a systematic and purposive way to create comparable groups of same-sex and different-sex married couples. Couples were first recruited using Massachusetts vital records to identify same-sex couples who had been married between 2004 and 2012 and met the age criteria. Initial recruitment began in Massachusetts because it was the first US state to legalize same-sex marriage, which enabled recruitment of a substantial number of long-term legally married same-sex couples. Although a portion of couples in the study were married in Massachusetts, they did not necessarily reside in Massachusetts at the time of recruitment and instead may have traveled to the state to legally marry or relocated following marriage. At the time of recruitment, 55% of same-sex male couples, 62% of same-sex female couples, and 51% of different-sex couples lived in states other than Massachusetts.

Different-sex couples were recruited by mailing letters to couples from zip codes corresponding to neighborhoods where significant numbers of lesbian and gay study participants lived. Potential respondents were identified through publicly available city lists that provided addresses and demographic information on household members. Participating couples were also asked to refer both same- and different-sex married couples in their social networks. About 70% of same-sex couples were recruited through vital statistics records; the remaining 30% were recruited via referrals. Approximately one-third of different-sex couples were recruited via city lists; the remaining two-thirds were recruited through referrals. For all study participants, invitation letters were sent to couples’ recorded addresses.

The HARP sample was designed to analyze midlife same-sex and different-sex married couples who were matched on age, relationship duration, and place of residence. Due to past legal restrictions on same-sex marriage, relationship duration was measured based on the combined number of years a couple had been cohabiting and married (i.e., total number of years partners had lived together). On average, respondents were 48 years old and had lived with their spouse for 15 years. Couples were socioeconomically advantaged: approximately half the sample had obtained a post-graduate degree and almost 60% reported a household income of $100,000 or more. Approximately 42% reported children living in their household, and 81% were working outside the home. Overall, the HARP sample was comparable to nationally representative data from midlife same- and different-sex couples in age, income, and percent of couples with children in the household (Gates 2015).

HARP data collection occurred in two parts. First, both spouses in each marriage completed an initial 30–40-min survey to obtain baseline data on demographics, relationship history, and mental and physical health. Participants then completed 10 consecutive nights of 5–10-min questionnaires. Spouses completed all surveys independently from each other and online. The analytic sample included couples who had completed the baseline survey and at least 6 of the daily diary questionnaires; 90% of participants completed all 10 days. Due to the data’s dyadic nature, both spouses had to have complete data to be included in the analytic sample. The final analytic sample included both spouses in 378 couples (*n* = 756 individuals): 157 women married to women couples (*n* = 314 women), 106 men married to men couples (*n* = 212 men), and 115 different-sex couples (*n* = 115 women; 115 men).

### 2.2 | Measures

#### 2.2.1 | Daily Psychological Distress

We measured daily psychological distress based on eight items from the daily diary survey that were adapted from instruments assessing negative affect and distress (Watson et al. 1988) with demonstrated reliability and validity (Crawford and Henry 2004). Respondents were asked: “Over the past 24 hours, to what extent did you feel: (1) calm, (2) happy, (3) frustrated, (4) worried, (5) sad, (6) irritable, (7) angry, (8) upset.” Each question had five response categories ranging from 1 (*Not at all)* to 5 (*Extremely*). Responses to these eight items were summed with item 1 and item 2 reverse coded (range: 8–40; alpha = 0.91).

#### 2.2.2 | Daily Stressor Exposure

Daily stressor exposure was measured by nine questions from the daily diary survey. Items were adapted from the Daily Inventory of Stressful Events (DISE; Almeida 2005; Almeida et al. 2002), modified for questionnaire administration (Totenhagen et al. 2012). Respondents were asked to indicate whether the following had occurred over the past 24 h (1 = “yes”): (1) “I had an argument with my spouse, (2) something happened with my spouse that I could have argued about but I decided to let it pass, (3) I had an argument or disagreement with someone other than my spouse, (4) housework and home demands, (5) work demands, (6) financial concerns, (7) health problem, (8) my spouse had some problem or difficulty, and (9) something else happened to me that most people would consider stressful.” Given low levels of missingness and in line with prior research (e.g., Totenhagen et al. 2012), we summed the nine items to create the measure of total *daily stressor exposure* (range 0–9; alpha = 0.62). Because spouses are interdependent (Carr and Springer 2010), researchers have called for the inclusion of stressors experienced by significant others (e.g., partners) when examining daily stress processes (Kiecolt-Glaser et al. 2020). Accordingly, the dyadic nature of the data allowed us to include an additional measure of total daily stressor exposure as independently reported by the respondent’s spouse, which we refer to as *daily spouse stressor exposure* (*r* = 0.39).

#### 2.2.3 | Covariates

In all analyses, we adjusted for sociodemographic and relationship factors likely associated with daily stress processes. Sociodemographic covariates were assessed in the baseline survey and included children present in the household (1 = “yes”), age (in years), educational attainment (less than a college degree (reference), a college degree, or a post-graduate degree), and employment status measured by whether the respondent was currently working outside of the home (1 = “yes”). We also accounted for relationship characteristics assessed in the baseline survey, including relationship duration (number of years married and cohabitating) and marital quality. Marital quality was measured using four items adapted from the Americans’ Changing Lives survey (ACL; House 2018). Respondents were asked: “In general, how much (1) does your spouse make you feel loved and cared for, (2) is your spouse willing to listen when you need to talk about your worries or problems, (3) is your spouse critical of you or what you do, and (4) does your spouse make too many demands on you?” Response options ranged from 1 (*Not at all*) to 5 (*A great deal*). Because positive and negative qualities of relationships are distinct and differentially influence health (Carr et al. 2016), we summed items 1 and 2 to create a measure of *marital support* (*r* = 0.57) and items 3 and 4 to assess *marital strain* (*r* = 0.60). Because approximately 90% of respondents identified as White, we did not include race/ethnicity as a covariate. Analyses included a control for *day of survey* (day 1–day 10) to help account for potential unmeasured effects related to time, such as habituation to the daily diary survey instrument (Bolger et al. 2003; Bolger and Laurenceau 2013). No additional measures beyond those detailed in the present section were assessed.

### 2.3 | Analytic Strategy

To assess differences in daily stress processes for women and men in same-sex and different-sex marriages, we used the factorial method (West et al. 2008)—an extension of the Actor-Partner Interdependence Model (APIM; Cook and Kenny 2005)—via mixed effects multilevel modeling with crossed random effects. Multilevel modeling can account for the nested structure of the data and the interdependence between spouses (Kenny and Kashy 2011; Kenny et al. 2006). Crossed random effects were necessary because partners and days are nested within couples but are “crossed” with one another (Kenny and Kashy 2011). We modeled the covariance structure for partners as exchangeable (constraining the error variances to be equal) to model the non-independence in the dyad (Kenny et al. 2006; West et al. 2008). This approach was designed for the analysis of non-independent dyadic data with indistinguishable (i.e., same-sex couples) and distinguishable (i.e., different-sex couples) dyads (Kenny et al. 2006; Kroeger and Powers 2019; West et al. 2008). The factorial method allowed for the examination of three gender effects: (1) respondent gender, (2) spouse gender, and (3) dyad gender. Dyad gender is assessed via an interaction between respondent gender and spouse gender that examines whether the effects of respondent and spouse gender are multiplicative. Together, the regression estimates can be used to calculate predicted scores for different gendered contexts of marriages: women married to women, women married to men, men married to women, and men married to men.

#### 2.3.1 | Daily Stressor Exposure

We first examined differences in daily stressor exposure for women and men in same-sex and different-sex couples. Model 1 tested whether respondent gender (1 = *woman*) and spouse gender (1 = *woman*) were associated with daily stressor exposure, controlling for spouse stressor exposure and individual- and couple-level factors. Model 2 added the interaction of respondent gender and spouse gender to examine whether and how these variables’ effects on daily stressor exposure differed when both spouses were women. We used estimates from Model 2 to calculate predicted daily stressor exposure scores for four groups: (1) women married to women, (2) women married to men, (3) men married to women, and (4) men married to men. We then tested for differences in daily stressor exposure across the four union types using post-estimation pairwise comparisons of the predicted scores.

#### 2.3.2 | Emotional Reactivity to Daily Stressors

We next examined whether daily stressors were associated with daily psychological distress and whether these within-person associations varied for women and men in same-sex and different-sex couples. Model 1 tested associations between daily stressor exposure and daily psychological distress for the full sample. Sequential models separately added the interaction of respondent gender and spouse gender (Model 2), the interaction of respondent gender and daily stressors to test whether the association between daily stressors and daily psychological distress differed for women and men (Model 3), and the interaction of spouse gender and daily stressors to test whether associations between daily stressors and daily psychological distress varied depending on whether the respondent was married to a woman or a man (Model 4). The final model included all interactions from preceding models as well as a three-way interaction of respondent gender, spouse gender, and daily stressors (Model 5). We used regression estimates from Model 5 to calculate predicted coefficients for the association between daily stressors and daily psychological distress for each couple type. Post-estimation pairwise comparisons of the predicted coefficients were used to test for differences in emotional reactivity across union types.

## 3 | Results

### 3.1 | Descriptive Statistics

Descriptive statistics for key variables are presented by union type in Table 1. To identify significant differences across union types, we used pairwise comparisons following unadjusted mixed effects multilevel models that account for the structure of the dyadic data. Regardless of whether they were in a same-sex or different-sex marriage, women reported higher daily psychological distress on average than men in same-sex marriages (women with women vs. men with men: *p* = 0.011; women with men vs. men with men: *p* < 0.001). Similar patterns were found for daily stressors (women with women vs. men with men: *p* = 0.002; women with men vs. men with men: *p* = 0.006). Women in different-sex marriages also reported higher daily psychological distress than men in different-sex marriages (*p* = 0.003).

Women and men in same-sex and different-sex marriages differed in their average levels of marital support and strain. Specifically, women and men in same-sex couples reported more marital support than women and men in different-sex couples (women with women vs. women with men: *p* < 0.001; women with women vs. men with women: *p* = 0.005; men with men vs. women with men: *p* = 0.004; men with men vs. men with women: *p* = 0.048). Conversely, men in different-sex marriages reported significantly more marital strain than all other couple types (*p* < 0.001). Relationship duration differed slightly across groups with women in same-sex marriages having shorter relationship durations than all other union types (vs. women with men and men with women: *p* = 0.025; vs. men with men: *p* = 0.011). A greater proportion of different-sex couples reported having children in the household compared to same-sex couples (*p* < 0.001). Among same-sex couples, a greater proportion of women reported children in the household than men (*p* < 0.001).

Demographically, same-sex couples were 3–4 years older than different-sex couples, on average (women with women vs. women with men: *p* < 0.001; women with women vs. men with women: *p* = 0.009; men with men vs. women with men: *p* < 0.001; men with men vs. men with women: *p* = 0.002). Women in different-sex couples were about 1.5 years younger on average than men in different-sex couples (*p* = 0.009). Women in same-sex couples were also more highly educated than different-sex couples. Specifically, a smaller proportion of women in same-sex couples attended some college or less compared to women (*p* = 0.018) and men in different-sex couples (*p* < 0.001). Conversely, a greater proportion of women in same-sex couples obtained a postgraduate degree compared to men in different-sex couples (*p* = 0.001). A greater proportion of women in different-sex couples also obtained a postgraduate degree compared to men in different-sex couples (*p* = 0.025). Employment status was similar across union types.

### 3.2 | Multivariate Analyses

#### 3.2.1 | Daily Stressor Exposure Across Union Types

We first explored whether daily stressor exposure differed for women and men in same-sex and different-sex marriages (Table 2). The significant positive coefficient for respondent gender in Model 1 indicates that, regardless of their spouse’s gender, women reported more daily stressors than men (*b* = 0.23; *p* = 0.002). The nonsignificant coefficient for spouse gender in Model 1 suggests that spouse gender was not associated with differences in exposure to daily stressors. Model 2 added an interaction between respondent gender and spouse gender. The nonsignificant interaction term suggests that the significant respondent gender effect in Model 1 was independent of spouse gender and that being married to a same-sex spouse was not associated with higher levels of stressor exposure.

**FIGURE 1 |:**
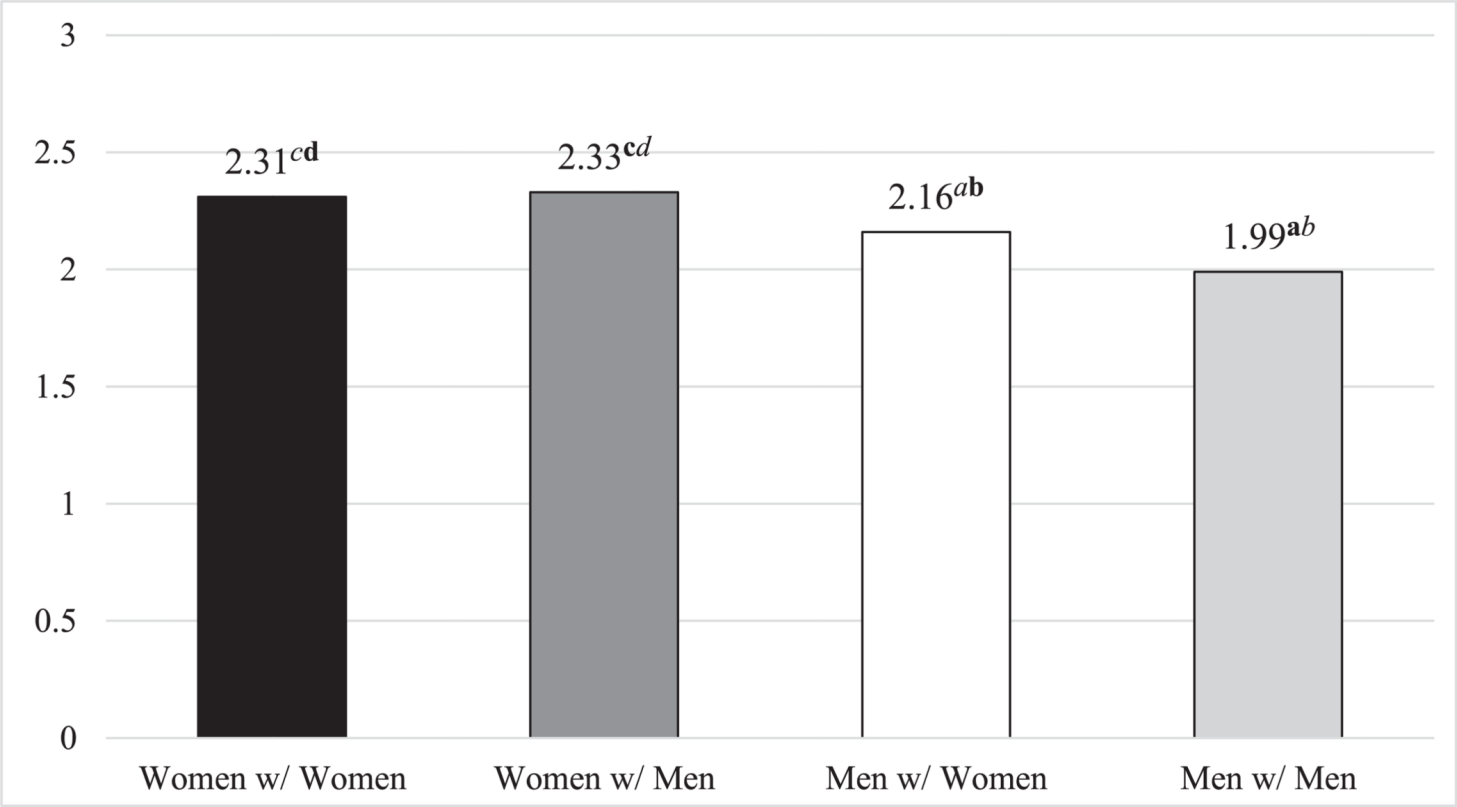
Predicted exposure to daily stressors by marital group. Estimates are derived from Model 2 of Table 2. Significant differences across groups are marked with superscripts in bold (*p* < 0.05) or italics (*p* < 0.10). ^a^Significantly different from women with women. ^b^Significantly different from women with men. ^c^Significantly different from men with women. ^d^Significantly different from men with men.

The inclusion of this interaction allowed us to estimate the predicted daily stressor exposure for each group (Figure 1). Post-estimation pairwise comparisons confirmed that women reported significantly more daily stressors than men across same-sex and different-sex marriages.

#### 3.2.2 | Emotional Reactivity to Daily Stressors

Next, we considered whether and how daily stressor exposure was associated with daily psychological distress for all couple types (Table 3, Models 1–2). To aid in interpretation, the outcome measure was standardized such that each coefficient represents the standard deviation change in daily psychological distress for a one-unit increase in the predictor. Model 1 shows a significant positive coefficient for daily stressor exposure, indicating that higher levels of daily stressor exposure were associated with higher levels of daily psychological distress for the full sample. Specifically, we found that each additional daily stressor was associated with a 0.20 standard deviation increase in daily psychological distress, net of individual- and couple-level factors (*p* < 0.001). The significant respondent gender coefficient in Model 1 indicates that women reported more daily psychological distress than men (*b* = 0.16; *p* < 0.001). Spouse gender was not significantly associated with daily psychological distress. In Model 2, the interaction term between respondent gender and spouse gender tested whether the respondent and spouse gender effects were independent when predicting daily psychological distress. The non-significant interaction term indicates that the significant respondent gender coefficient in Model 1 was independent of partner gender and dyad gender. Overall, results suggest that daily stressor exposure was significantly associated with daily psychological distress for the full sample.

#### 3.2.3 | Emotional Reactivity to Daily Stressors Across Union Types

To examine whether emotional reactivity to daily stressors differed for women and men in same-sex and different-sex marriages (Table 3), we interacted daily stressors with respondent gender (Model 3), spouse gender (Model 4), and respondent gender and spouse gender (Model 5). The results in Model 3 demonstrate that the association between daily stressors and daily psychological distress was stronger for women compared to men (*b* = 0.05; *p* = 0.001). Interestingly, the respondent gender effect found in Model 1 was no longer significant after including the interaction between daily stressors and respondent gender, which may indicate that gender differences in distress within marriage are explained by exposure *and* reactivity to daily stressors. In Model 4, the significant and negative interaction between daily stressors and spouse gender suggests a weaker association for respondents married to women compared to respondents married to men (*b* = −0.03; *p* = 0.021). Overall, results from Models 3–4 indicate being a woman strengthens the association between daily stressor exposure and daily psychological distress, whereas being married to a woman weakens the association.

Model 5 in Table 3 includes the three two-way interactions from Models 2–4 in addition to a three-way interaction of respondent gender, spouse gender, and respondent daily stressors. With the three-way interaction included in Model 5, the reference group becomes men married to men and is represented by the main effect for daily stressors. The interaction between respondent gender and daily stressors represents the added effect for women married to men, and the interaction between spouse gender and daily stressors represents the added effect for men married to women. Combining the previous two two-way interactions with the three-way interaction between respondent gender, spouse gender, and daily stressors represents the added effect for women married to women.

In Model 5, the interaction between respondent gender and daily stressors remains significant, indicating that the association between daily stressors and daily psychological distress was stronger for women married to men than for the reference group of men married to men (*b* = 0.06; *p* = 0.009). The interaction between spouse gender and daily stressors also remains significant, reflecting that this association was weaker for men married to women than for men married to men (*b* = −0.05; *p* = 0.016). The nonsignificant three-way interaction term indicates that this association did not differ once the genders of both spouses are accounted for. Taken together, results show that emotional reactivity to daily stressors was stronger for women in different-sex couples and weaker for men in different-sex couples compared to men in same-sex couples.

**FIGURE 2 |:**
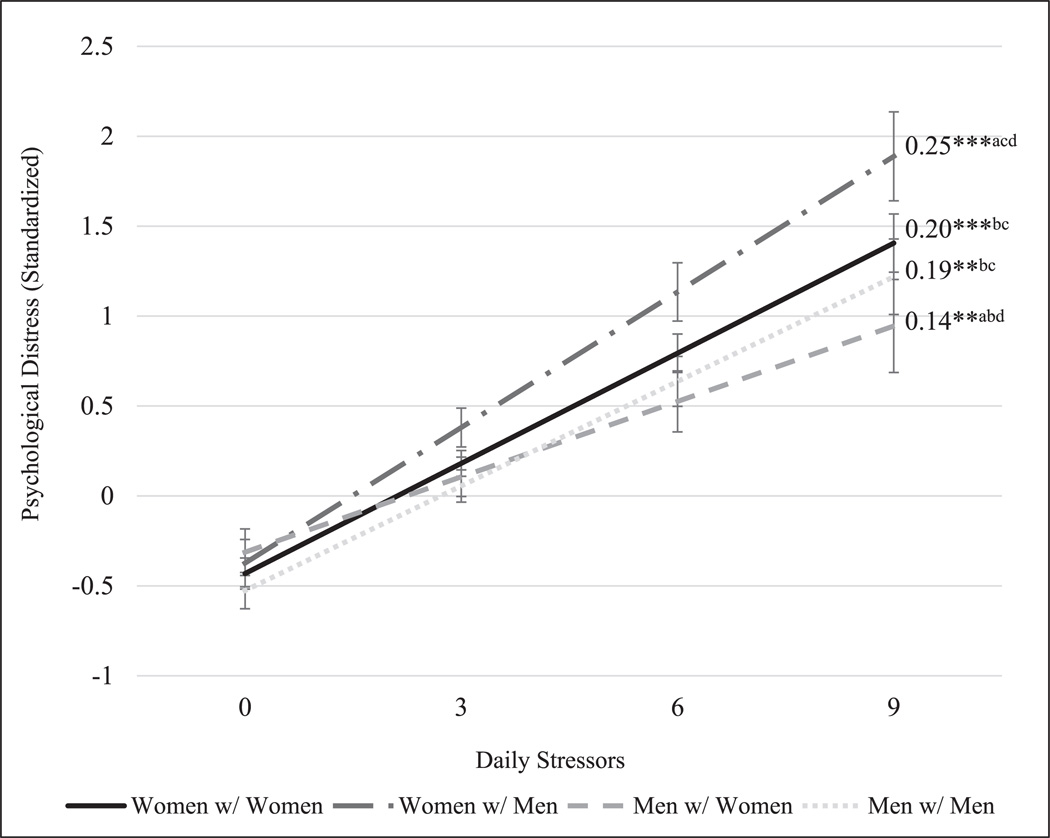
Adjusted predicted daily psychological distress by total daily stressor exposure. Estimates are derived from Model 5 of Table 3 and represent predicted daily psychological distress (standardized) as daily stressors increase. The slope coefficient for the overall association between total daily stressor exposure and daily psychological distress is presented by group (***p < 0.001, **p < 0.01) with significant group differences marked with superscripts (*p* < 0.05). ^a^Significantly different from women with women. ^b^Significantly different from women with men. ^c^Significantly different from men with women. dSignificantly different from men with men.

We used the results from Model 5 to calculate predicted coefficients for the association between daily stressors and daily psychological distress for each group. The association was significant for all groups, most strongly for women married to men (*b* = 0.25; *p* < 0.001), followed by women married to women (*b* = 0.20; *p* < 0.001), men married to men (*b* = 0.19; *p* < 0.001), and men married to women (*b* = 0.14; *p* < 0.001). Because the results of Model 5 only indicate significant differences from men married to men, we used post-estimation Wald tests to clarify whether the associations differed significantly across the other three groups. Results show that the association between daily stressors and daily psychological distress was significantly stronger for women married to men than for spouses in all other union types (vs. women with women: *p* = 0.021; vs. men with men: *p* = 0.009; vs. men with women: *p* < 0.001). Conversely, the association between daily stressors and daily psychological distress was weaker for men in different-sex couples than for men in same-sex couples (*p* = 0.016) and women in same-sex couples (*p* = 0.002). The association did not significantly differ for women and men in same-sex couples (Figure 2).

### 3.3 | Additional Analyses

#### 3.3.1 | Daily Stressor Exposure and Reactivity by Stressor Domains

Primary analyses consider total daily stressor exposure across stressor domains. We also considered whether exposure and reactivity differed for the nine specific stressors: (1) argument with spouse, (2) avoided argument with spouse, (3) argument with someone else, (4) housework/home demands, (5) work demands, (6) financial concerns, (7) health problem, (8) spouse had problem, (9) and other stressors. Considering stressor exposure by domain (Appendix S1: Table S1), results indicate significant differences across marital groups for exposure to housework/home demands, financial concerns, health problems, spouse problems, and other stressors. Specifically, women in same-sex couples reported housework and home demands more often than men in different-sex couples (*p* = 0.016). Men in same-sex couples reported financial concerns less often than women in same-sex (*p* = 0.010) and different-sex couples (*p* = 0.006). Conversely, women in different-sex couples reported health problems more often than men in same-sex (*p* = 0.009) and different-sex couples (*p* = 0.020). Women in same-sex couples reported spouse problems more often than men in same-sex couples (*p* < 0.001) and women in different-sex couples (*p* = 0.044). Finally, women in same-sex (*p* = 0.004) and different-sex couples (*p* = 0.033) reported other stressors more often than men in same-sex couples. Overall, results suggest that the higher exposure to daily stressors among women in same- and different-sex marriages is primarily driven by gender differences in housework/home demands, financial concerns, health problems, spouse problems, and other stressors, with some variation between women in same- and different-sex marriages.

Turning to emotional reactivity by stressor domain (Appendix S1: Table S2), results for the full sample indicate that each stressor domain was associated with increased psychological distress except for housework/home demands and spouse problems. We also considered differences in emotional reactivity to each stressor domain across the four marital groups. Results indicate that each stressor domain was significant across all four groups, with two exceptions. First, although housework/home demands were not significantly associated with psychological distress for the full sample, we find a significant and negative association for men in different-sex couples (*p* = 0.001). This is in line with recent research suggesting that increased shares of household labor improve well-being for husbands in different-sex marriages (Petts and Carlson 2023). Second, a spouse problem is associated with increased psychological distress for women in different-sex couples only (*p* = 0.001).

**FIGURE 3 |:**
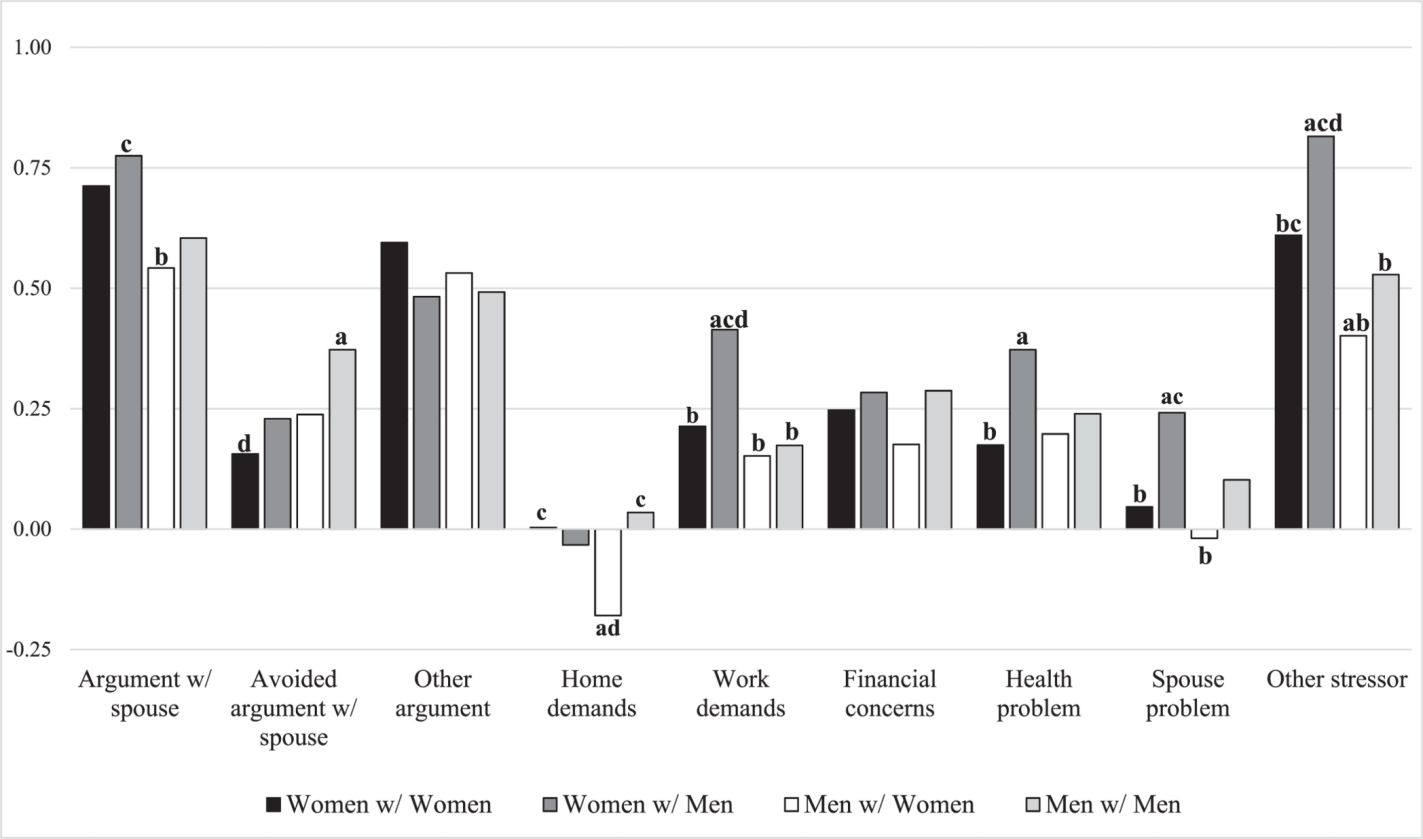
Adjusted predicted effect of daily stressor exposure on daily psychological distress by stressor domain. Predicted effect represents the standard deviation increase in daily psychological distress in the presence of a stressor. Significant differences across union types are marked by bolded letters (*p* < 0.05). ^a^Significantly different from women with women. ^b^Significantly different from women with men. ^c^Significantly different from men with women. dSignificantly different from men with men.

Concerning significant differences in emotional reactivity by stressor domain across union types (Figure 3), we find that arguments with a spouse were more strongly associated with daily psychological distress for women in different-sex couples compared to men in different-sex couples (*p* = 0.024). Conversely, the association for avoiding an argument with a spouse was significantly stronger for men in same-sex couples compared to women in same-sex couples (*p* = 0.002). The negative association between housework/home demands and psychological distress was significantly stronger for men in different-sex couples compared to women (*p* = 0.005) and men (*p* = 0.002) in same-sex couples. For work demands, the association was significantly stronger for women in different-sex couples compared to all other union types (vs. women with women: *p* = 0.002; vs. men with men: *p* = 0.001; vs. men with women: *p* < 0.001). The association between health problems and daily psychological distress was stronger for women in different-sex couples compared to women in same-sex couples (*p* = 0.011). The association for spouse problems was also stronger for women in different-sex couples compared to women in same-sex couples (*p* = 0.016) and men in different-sex couples (*p* = 0.007). Finally, the association between other stressors and psychological distress was significantly stronger for women in different-sex couples compared to all other union types (vs. women with women: *p* = 0.004; vs. men with men: *p* < 0.001; vs. men with women: *p* < 0.001) and for women in same-sex couples compared to men in different-sex couples (*p* = 0.005). As such, the greater emotional reactivity to daily stressors found among women married to men appears to be driven by their greater reactivity to arguments with a spouse, work demands, health problems, spouse problems, and other stressors.

#### 3.3.2 | Family Dynamics: Marital Quality and Children in the Household

Additional family dynamics may be important to consider when examining daily stress processes and well-being within marriage. Thus, additional analyses examined the potential moderating effects of marital quality (i.e., support and strain) and the presence of children in the household on emotional reactivity to daily stressors (Appendix S1: Table S3). Results indicate that both marital support and strain moderated the association between daily stressor exposure and daily psychological distress, but only for same-sex couples. That is, increased marital support reduced the association between daily stressor exposure and daily psychological distress for women married to women (*p* = 0.047) and men married to men (*p* < 0.001), whereas increased marital strain strengthened the association for women married to men (*p* = 0.008) and men married to men (*p* = 0.017). Having children present in the household strengthened the association between daily stressors and psychological distress, but only for women in different-sex marriages (*p* = 0.002).

## 4 | Discussion

Prior research suggests that women experience greater exposure (Villeneuve et al. 2014) and emotional reactivity (Bolger et al. 1989) to daily stressors than men and that such daily stress processes are important for understanding gender inequality in well-being in different-sex marriages (Almeida and Kessler 1998). However, gender differences in daily stress processes, and particularly emotional reactivity, have not been tested empirically in recent decades despite significant changes in gender dynamics related to work and home life that often result in an overload of stressors for women (Fry et al. 2023; Ridgeway 2011). Prior studies also focus exclusively on different- sex married couples, providing a limited view of how gender shapes daily stress processes in same-sex compared to different- sex marriages. The present study addresses these gaps by considering whether exposure and emotional reactivity to daily stressors differ for women and men in same-sex and different-sex marriages. Two major themes emerge from this study. First, women reported more daily stressors than men regardless of whether they were in a same-sex or a different-sex marriage. Second, emotional reactivity to daily stressors varied significantly by gender and union type: reactivity was greatest for women in different-sex marriages and least for men in different-sex marriages.

The first theme underscores the importance of gender for shaping exposure to daily stressors. In our sample, daily stressor exposure largely followed gender patterns established in prior research (Villeneuve et al. 2014) such that women in different-sex marriages reported more daily stressors than men in different-sex marriages. However, we go further to show that this gender difference is also true for women in same-sex marriages. That is, an individual’s gender seems to be the most salient predictor of exposure to daily stressors across couple types. Power differentials shape exposure to stress (Pearlin 2010; Thoits 2010), and our findings align with an understanding of gender as an enduring system of inequality that produces more daily demands for women than men regardless of marriage composition (Hill and Needham 2013; Read and Gorman 2010). For example, due to social expectations that women provide care within nuclear and extended families (Thomeer, Umberson, and Williams 2013), women in same- and different-sex marriages may experience additional stressors related to caring for children, aging parents, and/or friends. Although women in same-sex marriages tend to divide household and emotional labor more equitably (Goldberg et al. 2012; Tornello et al. 2015; Umberson et al. 2015, 2016), these benefits may be offset by their greater exposure to individual- and couple-level minority stressors (Cao et al. 2017; Frost et al. 2017; Meyer 2013).

Surprisingly, daily stressor exposure did not differ for men in same-sex marriages and different-sex marriages. This does not completely align with theories of individual- and couple-level minority stress (Cao et al. 2017; Frost et al. 2017; Meyer 2013) which suggest that men in same-sex marriages would be exposed to more daily stressors than men in different-sex marriages due to proximal (e.g., concealment) and distal (e.g., discrimination) social stressors related to their sexual orientation. Perhaps the gendered advantage of being a man and same-sex couples’ more equitable divisions of household labor help offset the burden of minority stressors faced by men in same-sex marriages, resulting in levels of daily stressor exposure that are similar to men in different-sex marriages. Men in different-sex marriages also reported more marital strain and less marital support compared to men in same-sex marriages, which may contribute to more marriage-based daily stressors among men married to women. Future research should incorporate measures of division of daily household labor and minority stressor exposure when examining variation in daily stress processes across same-sex and different-sex marriages.

The second major theme supports dyadic (Carr and Springer 2010) and gender-as-relational approaches (Connell 2012; Springer et al. 2012) to understanding gendered daily stress processes and well-being. Past research based on different-sex married couples suggests that emotional reactivity to daily stressors is stronger for women than for men (Bolger et al. 1989). We replicate this finding by showing that being a woman increases emotional reactivity to daily stressors, whereas being married to a woman decreases emotional reactivity. Importantly, we provide a much-needed update by showing that this pattern does not hold for same-sex married couples. This finding suggests a refined interpretation of the data: it is not that women are more reactive to daily stressors than men, but rather that women married to men experience greater reactivity to daily stressors than any other group, including not only men but also women married to women. Prior research helps explain this finding, as women in different-sex marriages tend to provide more and better-quality support to their spouse than they receive during times of stress (Neff and Karney 2005; Umberson et al. 2015), whereas spouses in same-sex marriages provide more equitable support to each other (Rostosky and Riggle 2017; Umberson et al. 2015, 2016). However, future research should aim to identify the specific mechanisms linking daily stressors with emotional reactivity.

We also found that women and men in same-sex couples were more emotionally reactive to daily stressors than men in different-sex marriages. This suggests that women in same-sex marriages may be disadvantaged due to their own gender but benefit from being married to a woman, whereas men in same-sex couples benefit from their gender but are disadvantaged by being married to a man. Same-sex couples also carry the additional burden of coping with minority stressors together (Cao et al. 2017; Frost et al. 2017; Meyer 2013), which could contribute to their increased emotional reactivity relative to men in different-sex marriages.

We note several limitations of the study. First, the measure of daily stressors used a count of the total number of stressors experienced in a day but does not consider stressor severity, which may correspond to differences in reactivity across union types; future research could incorporate such measures when possible. Second, some participating couples were recruited using purposive sampling, which could lead to non-independent measures. Because some different-sex couples were recruited by identifying neighborhoods with substantial numbers of same-sex couples, they may be more progressive and egalitarian regarding gendered divisions of labor and support exchanges than different-sex couples on average. Thus, the differences in daily stressor exposure and emotional reactivity found in the present study may underestimate differences between same-sex and different-sex married couples.

Third, the sample is also relatively privileged in terms of socioeconomic status, race/ethnicity, and gender identity (i.e., cisgender). Less educated individuals, racial/ethnic minorities, and gender minorities are exposed to more stressors and are disadvantaged in terms of stress-ameliorating resources such as money, power, and prestige (Meyer 2013; Pearlin 2010). Future research would benefit from an intersectional perspective (Collins 2019) to consider the ways in which stress processes and well-being are shaped by structures of power across multiple social statuses. It is also important to note the historical context when data collection occurred and the uniqueness of this cohort of married couples. Because same-sex couples in the present study were married prior to the nationwide legalization of same-sex marriage in 2015, they may represent a cohort of same-sex couples that faced unique challenges by being married during a time when access to marriage was relatively limited. Thus, future research should consider whether the patterns found in the present study differ over time and across birth cohorts. Despite these limitations, this study is an important first step toward understanding gendered daily stress processes across both same-sex and different-sex marriages.

This study provides new insights into linkages between gendered daily stress processes and well-being for midlife women and men in same-sex and different-sex marriages. Our findings suggest that women in both same-sex and different-sex marriages are exposed to more daily stressors than men and that women in different-sex marriages are uniquely disadvantaged by this exposure via heightened emotional reactivity. These findings reinforce evidence that exposure to daily stressors has direct and immediate impacts on well-being, underscore the importance of including same-sex couples in research on gendered marital dynamics, and highlight the need to consider the gender of each spouse in research on daily stress processes and well-being in same-sex and different-sex marriages. Considering the heightened risk of stress and poorer mental health profiles of women (Thoits 2010) and sexual minority populations (Meyer 2013; National Academies of Sciences, Engineering, and Medicine 2020), this study has the potential to inform future research aimed at promoting health equity. Findings shed light on who is most at risk of daily stressors and may help couples, clinicians, and other stakeholders understand how to mitigate the adverse consequences of daily stressors. For example, clinicians might focus more on addressing the gender gap in exposure and emotional reactivity to daily stressors within different-sex couples, but they might focus more on vulnerabilities associated with high stress levels among both spouses in female couples. The present findings can help clinicians and other providers support couples as they navigate daily stressors, especially spouses in same-sex marriages for whom there is far less research on evidence-based interventions.

## Supplementary Material

Supplemental Tables **Table S1:** Odds ratios from mixed effects logistic regression models predicting daily stressor exposure by stressor domain (*n* = 756 individuals, 378 couples). Odds ratios from mixed effects logistic regression models predicting individual daily stressor exposure by stressor domain (*n* = 756 individuals, 378 couples)
**Table S2:** Estimates from multi-level regression models predicting daily psychological distress by individual daily stressor exposure (*n* = 756 individuals, 378 couples). Estimates from multi-level regression models predicting daily psychological distress by individual daily stressor exposure (*n* = 756 individuals, 378 couples)
**Table S3:** Estimates from multi-level regression models predicting moderation effects on the association between daily stressor exposure and daily psychological distress (*n* = 756 individuals, 378 couples).

Additional supporting information can be found online in the Supporting Information section.

## Figures and Tables

**TABLE 1 |:** Descripive statistics by gender composition of the couple (*n* = 756 individuals; 378 couples).

Variables	Women with women (*n* = 314)		Women with men (*n* = 115)		Men with women (*n* = 115)		Men with men (*n* = 212)	
	*M*	SD	*M*	SD	*M*	SD	*M*	SD

Psychological distress	14.33^d^	(3.43)	15.07^c,d^	(3.38)	14.04^b^	(3.11)	13.47^a,b^	(3.14)
Daily stressors (total)	2.31^d^	(1.05)	2.34^d^	(1.11)	2.17	(0.98)	1.98^a,b^	(1.00)
Marital support	6.81^b,c^	(1.38)	6.15^a,d^	(1.86)	6.32^a,d^	(1.68)	6.70^b,c^	(1.48)
Marital strain	1.86^c^	(1.68)	1.85^c^	(1.64)	2.63^a,b,d^	(1.80)	1.77^c^	(1.65)
Relationship duration (years)	13.73^b,c,d^	(7.69)	15.89^a^	(8.18)	15.89^a^	(8.18)	16.23^a^	(7.79)
Children in household (% yes)	40.45^b,c,d^		71.30^a,d^		71.30^a,d^		12.26^a,b,c^	
Age (years)	49.04^b,c^	(8.38)	45.04^a,c,d^	(7.62)	46.48^a,b,d^	(8.11)	49.72^b,c^	(8.46)
Education (%)								
Some college or less	13.69^a,c^		24.35^a^		30.43^a^		19.81	
College degree	28.98		26.09		32.17		31.13	
Postgraduate degree	57.32^c^		49.57^c^		37.39^a,b^		49.06	
Currently working (% yes)	82.80		76.52		79.82		83.02	

*Note:* Differences from post hoc pairwise comparison following unadjusted mixed effects multilevel models (*p* < 0.05).

a Significantly different from women with women.

b Significantly different from women with men.

c Significantly different from men with women.

d Significantly different from men with men.

**TABLE 2 |:** Estimates from multi-level regression models predicting daily stressor exposure (*n* = 756 individuals, 378 couples).

Variables	Model 1		Model 2	
	*B*	(SE)	*B*	(SE)

Respondent woman	0.23**	(0.07)	0.26	(0.13)
Spouse woman	0.01	(0.08)	0.04	(0.13)
Respondent woman × spouse woman			−0.05	(0.20)
Spouse daily stressors (total: 0–9)	0.01	(0.01)	0.01	(0.01)
Marital support (range: 1–10)	−0.10***	(0.03)	−0.10***	(0.03)
Marital strain (range: 1–10)	0.08***	(0.02)	0.08***	(0.02)
Relationship duration (years)	−0.01	(0.01)	−0.01	(0.01)
Children in household (1 = yes)	0.15	(0.09)	0.14	(0.10)
Age (years)	0.01	(0.01)	0.01	(0.01)
College degree (ref. some college or less)	0.37***	(0.10)	0.37***	(0.10)
Post-grad degree (ref. some college or less)	0.34***	(0.10)	0.34***	(0.10)
Currently working (1 = yes)	0.11	(0.09)	0.11	(0.09)
Day of survey (1–10)	−0.04***	(0.01)	−0.04***	(0.01)
Constant	2.12***	(0.35)	2.11***	(0.36)

*Note:* Standard errors in parentheses.

** *p* < 0.01.

*** *p* < 0.001.

**TABLE 3 |:** Estimates from multi-level regression models predicting daily psychological distress by daily stressor exposure (*n* = 756 individuals, 378 couples).

Variables	Model 1		Model 2		Model 3		Model 4		Model 5	
	*B*	(SE)	*B*	(SE)	*B*	(SE)	*B*	(SE)	*B*	(SE)

Daily stressors (total: 0–9)	0.20***	(0.01)	0.20***	(0.01)	0.17***	(0.01)	0.22***	(0.01)	0.19***	(0.01)
Respondent (R) woman	0.16***	(0.05)	0.20**	(0.08)	0.06	(0.06)	0.16***	(0.05)	0.07	(0.09)
Spouse (S) woman	−0.06	(0.05)	−0.02	(0.08)	−0.05	(0.05)	0.02	(0.06)	0.10	(0.09)
Respondent woman × spouse woman			−0.08	(0.12)					−0.09	(0.14)
Respondent woman × daily stressors					0.05**	(0.01)			0.06**	(0.02)
Spouse woman × daily stressors							−0.03*	(0.01)	−0.05*	(0.02)
R woman × S woman × daily stressors									0.01	(0.03)
Spouse daily stressors (total: 0–9)	0.00	(0.01)	0.00	(0.01)	0.00	(0.01)	0.00	(0.01)	0.00	(0.01)
Marital support (range: 1–10)	−0.07***	(0.02)	−0.07***	(0.02)	−0.07***	(0.02)	−0.07***	(0.02)	−0.07***	(0.02)
Marital strain (range: 1–10)	0.06***	(0.01)	0.06***	(0.01)	0.06***	(0.01)	0.06***	(0.01)	0.06***	(0.01)
Relationship duration (years)	−0.00	(0.00)	−0.00	(0.00)	−0.00	(0.00)	−0.00	(0.00)	−0.00	(0.00)
Children in household (1 = yes)	0.03	(0.05)	0.01	(0.06)	0.03	(0.05)	0.03	(0.05)	0.01	(0.06)
Age (years)	−0.01	(0.00)	−0.01	(0.00)	−0.01	(0.00)	−0.01	(0.00)	−0.01	(0.00)
College degree (ref. some college or less)	0.12	(0.06)	0.12	(0.06)	0.12	(0.06)	0.12	(0.06)	0.12*	(0.06)
Post-grad degree (ref. some college or less)	0.00	(0.06)	0.01	(0.06)	0.00	(0.06)	0.00	(0.06)	0.01	(0.06)
Currently working (1 = yes)	−0.14*	(0.06)	−0.14*	(0.06)	−0.14*	(0.06)	−0.14*	(0.06)	−0.14*	(0.06)
Day of survey (1–10)	−0.01	(0.00)	−0.01	(0.00)	−0.01	(0.00)	−0.01	(0.00)	−0.01*	(0.00)
Constant	0.30	(0.21)	0.27	(0.21)	0.35	(0.21)	0.24	(0.21)	0.27	(0.21)

*Note:* Standard errors in parentheses. Outcome is standardized to aid in interpretation (i.e., coefficients represent the standard deviation change in psychological distress for a one-unit increase in the predictor).

* *p* < 0.05.

** *p* < 0.01.

*** *p* < 0.001.

## Data Availability

The data that support the findings of this study are openly available through ICPSR at https://doi.org/10.3886/ICPSR37404.v2.
