# Supplementary material for: Exposure and Emotional Reactivity to Daily Stressors in Same-Sex and Different-Sex Marriages
Source: J Marriage Fam. Author manuscript; Available in PMC 2026 Apr 9. (PMC13061512; doi:10.1111/jomf.70058)
Supplement: Supplemental Tables — Table S1: Odds ratios from mixed effects logistic regression models predicting daily stressor exposure by stressor domain (n = 756 individuals, 378 couples). Odds ratios from mixed effects logistic regression models predicting individual daily stressor exposure by stressor domain (n = 756 individuals, 378 couples) Table S2: Estimates from multi-level regression models predicting daily psychological distress by individual daily stressor exposure (n = 756 individuals, 378 couples). Estimates from multi-level regression models predicting daily psychological distress by individual daily stressor exposure (n = 756 individuals, 378 couples) Table S3: Estimates from multi-level regression models predicting moderation effects on the association between daily stressor exposure and daily psychological distress (n = 756 individuals, 378 couples). [file NIHMS2150188-supplement-Supplemental_Tables.docx]

**Online Only Appendix: Exposure and Emotional Reactivity to Daily Stressors in Same-Sex and Different-Sex Marriages**Authors: Michael A. Garcia, Rachel Donnelly, and Debra Umberson

| Table S1. *Odds Ratios from Mixed Effects Logistic Regression Models Predicting Daily Stressor Exposure by Stressor Domain (n = 756 individuals, 378 couples)* | | | | | | | | | | |
| --- | --- | --- | --- | --- | --- | --- | --- | --- | --- | --- |
|  | Argument with spouse | | Avoided argument with spouse | | Argument with someone else | | Housework/  home demands | | Work  demands | |
| Variables | *B* | *(SE)* | *B* | *(SE)* | *B* | *(SE)* | *B* | *(SE)* | *B* | *(SE)* |
| Respondent woman | 1.19 | (0.23) | 1.18 | (0.23) | 0.98 | (0.23) | 1.04 | (0.27) | 0.84 | (0.17) |
| Spouse woman | 0.99 | (0.19) | 1.07 | (0.20) | 0.66 | (0.16) | 0.69 | (0.18) | 1.20 | (0.24) |
| Respondent woman × Spouse woman | 1.04 | (0.28) | 0.79 | (0.21) | 1.35 | (0.46) | 1.70 | (0.63) | 1.10 | (0.31) |
|  |  |  |  |  |  |  |  |  |  |  |
| Spouse daily stressors (total: 0-9) | 1.08* | (0.03) | 1.08** | (0.03) | 1.12*** | (0.04) | 0.99 | (0.03) | 1.04 | (0.02) |
| Marital support (range: 1-10) | 0.93 | (0.03) | 0.88** | (0.03) | 0.93 | (0.05) | 0.92 | (0.05) | 0.94 | (0.04) |
| Marital strain (range: 1-10) | 1.24*** | (0.04) | 1.27*** | (0.04) | 1.11* | (0.05) | 1.05 | (0.05) | 1.06 | (0.04) |
| Relationship duration (years) | 0.98* | (0.01) | 0.98** | (0.01) | 1.00 | (0.01) | 1.01 | (0.01) | 1.00 | (0.01) |
| Children in household (1 = yes) | 1.21 | (0.16) | 1.13 | (0.15) | 1.45* | (0.25) | 2.05*** | (0.38) | 0.76 | (0.11) |
| Age (years) | 1.00 | (0.01) | 1.01 | (0.01) | 1.00 | (0.01) | 1.00 | (0.01) | 1.00 | (0.01) |
| College degree (ref. some college or less) | 1.08 | (0.18) | 1.14 | (0.19) | 0.99 | (0.20) | 2.66*** | (0.58) | 1.72** | (0.29) |
| Post-grad degree (ref. some college or less) | 1.10 | (0.17) | 1.13 | (0.17) | 1.03 | (0.19) | 2.81*** | (0.57) | 1.79*** | (0.28) |
| Currently working (1 = yes) | 1.03 | (0.15) | 0.79 | (0.11) | 1.26 | (0.24) | 0.62* | (0.12) | 13.18*** | (2.27) |
| Day of survey (1-10) | 0.98 | (0.01) | 0.97* | (0.01) | 0.95*** | (0.01) | 0.95*** | (0.01) | 0.96*** | (0.01) |
| Constant | 0.06*** | (0.03) | 0.14*** | (0.07) | 0.05*** | (0.04) | 1.35 | (0.96) | 0.11*** | (0.06) |
| Note: Standard errors in parentheses. Estimates derived from the final model of a two-model series using the factorial method. Model 1 results available upon request. ****p* < .001, ***p* < .01, **p* < .05. | | | | | | | | | | |

| Table S1 (cont.). *Odds Ratios from Mixed Effects Logistic Regression Models Predicting Individual Daily Stressor Exposure by Stressor Domain (n = 756 individuals, 378 couples)* | | | | | | | | |
| --- | --- | --- | --- | --- | --- | --- | --- | --- |
|  | Financial concerns | | Health problem | | Spouse problem | | Other  stressor | |
| Variables | *B* | *(SE)* | *B* | *(SE)* | *B* | *(SE)* | *B* | *(SE)* |
| Respondent woman | 2.59** | (0.89) | 2.32** | (0.74) | 1.26 | (0.28) | 1.51* | (0.30) |
| Spouse woman | 1.83 | (0.63) | 1.05 | (0.34) | 1.52 | (0.33) | 1.13 | (0.22) |
| Respondent woman × Spouse woman | 0.41 | (0.20) | 0.59 | (0.27) | 0.99 | (0.31) | 0.89 | (0.25) |
|  |  |  |  |  |  |  |  |  |
| Spouse daily stressors (total: 0-9) | 1.11*** | (0.03) | 1.05 | (0.03) | 1.07* | (0.03) | 1.11*** | (0.03) |
| Marital support (range: 1-10) | 0.78*** | (0.06) | 0.86* | (0.06) | 0.91* | (0.04) | 0.91* | (0.04) |
| Marital strain (range: 1-10) | 1.16* | (0.08) | 0.96 | (0.06) | 0.98 | (0.04) | 1.03 | (0.04) |
| Relationship duration (years) | 0.98 | (0.02) | 0.98 | (0.01) | 1.00 | (0.01) | 0.98* | (0.01) |
| Children in household (1 = yes) | 1.26 | (0.31) | 0.84 | (0.19) | 0.92 | (0.14) | 0.87 | (0.12) |
| Age (years) | 0.99 | (0.02) | 1.04* | (0.02) | 1.01 | (0.01) | 1.02* | (0.01) |
| College degree (ref. some college or less) | 2.05* | (0.60) | 1.57 | (0.43) | 1.00 | (0.19) | 1.16 | (0.19) |
| Post-grad degree (ref. some college or less) | 1.33 | (0.36) | 1.34 | (0.34) | 1.19 | (0.20) | 1.15 | (0.18) |
| Currently working (1 = yes) | 0.40*** | (0.10) | 0.48** | (0.12) | 0.73 | (0.12) | 1.11 | (0.17) |
| Day of survey (1-10) | 0.97* | (0.01) | 0.92*** | (0.01) | 0.92*** | (0.01) | 0.95*** | (0.01) |
| Constant | 0.80 | (0.76) | 0.12* | (0.10) | 0.13*** | (0.08) | 0.09*** | (0.05) |
| Note: Standard errors in parentheses. Estimates derived from the final model of a two-model series using the factorial method. Model 1 results available upon request. ****p* < .001, ***p* < .01, **p* < .05. | | | | | | | | |

| Table S2. *Estimates from Multi-Level Regression Models Predicting Daily Psychological Distress by Individual Daily Stressor Exposure (n = 756 individuals, 378 couples)* | | | | | | | | | | |
| --- | --- | --- | --- | --- | --- | --- | --- | --- | --- | --- |
|  | Argument with spouse | | Avoided argument with spouse | | Argument with someone else | | Housework/  home demands | | Work  demands | |
| Variables | *B* | *(SE)* | *B* | *(SE)* | *B* | *(SE)* | *B* | *(SE)* | *B* | *(SE)* |
| Daily stressor (1 = yes) | 0.60*** | (0.07) | 0.37*** | (0.05) | 0.49*** | (0.06) | 0.03 | (0.04) | 0.17*** | (0.04) |
| Respondent (R) woman | 0.23** | (0.08) | 0.27** | (0.09) | 0.25** | (0.08) | 0.30** | (0.10) | 0.15 | (0.09) |
| Spouse (S) woman | -0.00 | (0.08) | 0.01 | (0.09) | 0.00 | (0.08) | 0.11 | (0.09) | -0.00 | (0.09) |
| Respondent woman × Spouse woman | -0.09 | (0.13) | -0.09 | (0.13) | -0.10 | (0.13) | -0.23 | (0.14) | -0.01 | (0.14) |
| Respondent woman × Daily stressor | 0.17 | (0.10) | -0.14 | (0.09) | -0.01 | (0.10) | -0.07 | (0.07) | 0.24*** | (0.07) |
| Spouse woman × Daily stressor | -0.06 | (0.10) | -0.13 | (0.08) | 0.04 | (0.10) | -0.21** | (0.07) | -0.02 | (0.07) |
| R woman × S woman × Daily stressor | -0.00 | (0.15) | 0.06 | (0.12) | 0.07 | (0.14) | 0.25** | (0.09) | -0.18 | (0.09) |
|  |  |  |  |  |  |  |  |  |  |  |
| Spouse daily stressors (total: 0-9) | 0.00 | (0.01) | 0.00 | (0.01) | -0.00 | (0.01) | 0.00 | (0.01) | 0.00 | (0.01) |
| Marital support (range: 1-10) | -0.09*** | (0.02) | -0.08*** | (0.02) | -0.09*** | (0.02) | -0.09*** | (0.02) | -0.09*** | (0.02) |
| Marital strain (range: 1-10) | 0.06*** | (0.01) | 0.07*** | (0.01) | 0.07*** | (0.01) | 0.07*** | (0.01) | 0.07*** | (0.01) |
| Relationship duration (years) | -0.00 | (0.00) | -0.00 | (0.00) | -0.00 | (0.00) | -0.00 | (0.00) | -0.00 | (0.00) |
| Children in household (1 = yes) | 0.03 | (0.06) | 0.04 | (0.06) | 0.03 | (0.06) | 0.04 | (0.07) | 0.06 | (0.06) |
| Age (years) | -0.01 | (0.00) | -0.00 | (0.00) | -0.00 | (0.00) | -0.00 | (0.00) | -0.01 | (0.00) |
| College degree (ref. some college or less) | 0.20** | (0.07) | 0.19** | (0.07) | 0.20** | (0.07) | 0.20** | (0.07) | 0.17** | (0.07) |
| Post-grad degree (ref. some college or less) | 0.07 | (0.06) | 0.07 | (0.06) | 0.08 | (0.06) | 0.08 | (0.06) | 0.05 | (0.06) |
| Currently working (1 = yes) | -0.12* | (0.06) | -0.11 | (0.06) | -0.13* | (0.06) | -0.11 | (0.06) | -0.21*** | (0.06) |
| Day of survey (1-10) | -0.01*** | (0.00) | -0.01*** | (0.00) | -0.01*** | (0.00) | -0.02*** | (0.00) | -0.01*** | (0.00) |
| Constant | 0.63** | (0.23) | 0.61** | (0.23) | 0.64** | (0.23) | 0.65** | (0.23) | 0.68** | (0.23) |
| Note: Standard errors in parentheses. Outcome is standardized to aid in interpretation (i.e., coefficients represent the standard deviation change in psychological distress for a one-unit increase in the predictor). Estimates derived from the final model of a five-model series using the factorial method. Full results for Models 1-4 are available upon request. ****p* < .001, ***p* < .01, **p* < .05. | | | | | | | | | | |

| Table S2 (cont.). *Estimates from Multi-Level Regression Models Predicting Daily Psychological Distress by Individual Daily Stressor Exposure (n = 756 individuals, 378 couples)* | | | | | | | | |
| --- | --- | --- | --- | --- | --- | --- | --- | --- |
|  | Financial concerns | | Health problem | | Spouse problem | | Other  stressor | |
| Variables | *B* | *(SE)* | *B* | *(SE)* | *B* | *(SE)* | *B* | *(SE)* |
| Daily stressor (1 = yes) | 0.29*** | (0.05) | 0.24*** | (0.05) | 0.10 | (0.05) | 0.53*** | (0.05) |
| Respondent (R) woman | 0.22* | (0.09) | 0.21* | (0.09) | 0.22* | (0.09) | 0.16 | (0.08) |
| Spouse (S) woman | 0.01 | (0.09) | 0.00 | (0.09) | 0.01 | (0.09) | 0.01 | (0.08) |
| Respondent woman × Spouse woman | -0.08 | (0.13) | -0.05 | (0.13) | -0.07 | (0.13) | -0.06 | (0.12) |
| Respondent woman × Daily stressor | -0.00 | (0.08) | 0.13 | (0.08) | 0.14 | (0.09) | 0.29*** | (0.08) |
| Spouse woman × Daily stressor | -0.11 | (0.08) | -0.04 | (0.09) | -0.12 | (0.09) | -0.13 | (0.08) |
| R woman × S woman × Daily stressor | 0.07 | (0.11) | -0.16 | (0.12) | -0.07 | (0.12) | -0.08 | (0.11) |
|  |  |  |  |  |  |  |  |  |
| Spouse daily stressors (total: 0-9) | 0.00 | (0.01) | 0.00 | (0.01) | 0.00 | (0.01) | -0.00 | (0.01) |
| Marital support (range: 1-10) | -0.08*** | (0.02) | -0.09*** | (0.02) | -0.09*** | (0.02) | -0.08*** | (0.02) |
| Marital strain (range: 1-10) | 0.07*** | (0.01) | 0.07*** | (0.01) | 0.07*** | (0.01) | 0.07*** | (0.01) |
| Relationship duration (years) | -0.00 | (0.00) | -0.00 | (0.00) | -0.00 | (0.00) | -0.00 | (0.00) |
| Children in household (1 = yes) | 0.04 | (0.06) | 0.04 | (0.06) | 0.05 | (0.06) | 0.06 | (0.06) |
| Age (years) | -0.00 | (0.00) | -0.01 | (0.00) | -0.00 | (0.00) | -0.01 | (0.00) |
| College degree (ref. some college or less) | 0.18** | (0.07) | 0.19** | (0.07) | 0.19** | (0.07) | 0.19** | (0.06) |
| Post-grad degree (ref. some college or less) | 0.07 | (0.06) | 0.07 | (0.06) | 0.07 | (0.06) | 0.07 | (0.06) |
| Currently working (1 = yes) | -0.10 | (0.06) | -0.11 | (0.06) | -0.12* | (0.06) | -0.13* | (0.06) |
| Day of survey (1-10) | -0.01*** | (0.00) | -0.01*** | (0.00) | -0.01*** | (0.00) | -0.01** | (0.00) |
| Constant | 0.54* | (0.22) | 0.67** | (0.23) | 0.65** | (0.23) | 0.57** | (0.22) |
| Note: Standard errors in parentheses. Outcome is standardized to aid in interpretation (i.e., coefficients represent the standard deviation change in psychological distress for a one-unit increase in the predictor). Estimates derived from the final model of a five-model series using the factorial method. Full results for Models 1-4 are available upon request. ****p* < .001, ***p* < .01, **p* < .05. | | | | | | | | |

| Table S3. *Estimates from Multi-Level Regression Models Predicting Moderation Effects on the Association Between*  *Daily Stressor Exposure and Daily Psychological Distress (n = 756 individuals, 378 couples)* | | | | | | |
| --- | --- | --- | --- | --- | --- | --- |
|  | Marital support | | Marital strain | | Children in household | |
| Variables | *B* | *(SE)* | *B* | *(SE)* | *B* | *(SE)* |
| Daily stressors (total: 0-9) | 0.42*** | (0.06) | 0.16*** | (0.02) | 0.19*** | (0.01) |
| Respondent (R) woman | 0.14 | (0.35) | 0.14 | (0.13) | 0.26 | (0.14) |
| Spouse (S) woman | 0.40 | (0.36) | 0.23 | (0.14) | 0.22 | (0.14) |
| *Moderators* |  |  |  |  |  |  |
| Marital support (range: 1-10) | 0.00 | (0.04) |  |  |  |  |
| Marital strain (range: 1-10) |  |  | 0.05 | (0.03) |  |  |
| Children in household (1 = yes) |  |  |  |  | 0.12 | (0.16) |
| Respondent woman × Spouse woman | 0.01 | (0.49) | -0.21 | (0.20) | -0.38 | (0.21) |
| Respondent woman × Daily stressors | -0.15 | (0.08) | 0.09** | (0.03) | -0.02 | (0.03) |
| Respondent woman × Moderator | -0.01 | (0.05) | -0.04 | (0.05) | -0.36 | (0.22) |
| Spouse woman × Daily stressors | -0.25** | (0.09) | -0.06 | (0.04) | -0.08* | (0.04) |
| Spouse woman × Moderator | -0.04 | (0.05) | -0.05 | (0.05) | -0.25 | (0.22) |
| Daily stressors × Moderator | -0.03*** | (0.01) | 0.02* | (0.01) | -0.00 | (0.04) |
| R woman × S woman × Daily stressors | 0.28* | (0.12) | -0.01 | (0.05) | 0.12* | (0.05) |
| R woman × S woman × Moderator | -0.02 | (0.07) | 0.05 | (0.07) | 0.50 | (0.29) |
| R woman × Daily stressors × Moderator | 0.03* | (0.01) | -0.02 | (0.01) | 0.12* | (0.06) |
| S woman × Daily stressors × Moderator | 0.03* | (0.01) | -0.00 | (0.01) | 0.03 | (0.06) |
| R woman × S woman × Daily stressors × Moderator | -0.04* | (0.02) | 0.02 | (0.02) | -0.16* | (0.07) |
| Constant | -0.19 | (0.31) | 0.30 | (0.22) | 0.28 | (0.21) |
| Note: Standard errors in parentheses. Outcome is standardized to aid in interpretation (i.e., coefficients represent the standard deviation change in psychological distress for a one-unit increase in the predictor). Estimates derived from the final model of a five-model series using the factorial method. Covariates for spouse daily stressors, relationship duration, age, education, employment status, and day of survey are included but not shown. Full results for Models 1-4 are available upon request. ****p* < .001, ***p* < .01, **p* < .05. | | | | | | |
